# Hemoglobin Concentration and Post-Operative Delirium in Elderly Patients Undergoing Femoral Neck Fracture Surgery

**DOI:** 10.3389/fmed.2021.780196

**Published:** 2022-01-05

**Authors:** Yu-Mei Liu, Hui Huang, Jie Gao, Jian Zhou, Hai-Chen Chu

**Affiliations:** ^1^School of Clinical Medicine, Qingdao University, Qingdao, China; ^2^Department of Anesthesiology, The Affiliated Hospital of Qingdao University, School of Clinical Medicine, Qingdao University, Qingdao, China; ^3^West China School of Medicine, West China Hospital of Sichuan University, Chengdu, China

**Keywords:** delirium, old age, hemoglobin, risk factors, perioperation

## Abstract

This study aimed to determine the relationship between hemoglobin (Hb) concentration and post-operative delirium (POD) in elderly patients undergoing femoral neck fracture (FNF) surgery and to investigate whether the change in Hb concentration is associated with POD and the risk factors for POD. A total of 889 patients admitted with FNF between January 2016 and December 2020 were enrolled in this single-center, retrospective, case–control study. Hb concentrations were determined at admission and post-operative day 1 and the change in Hb concentration was defined as the absolute value of difference in pre-operative and post-operative Hb concentration. POD was assessed using the Confusion Assessment Method for the Intensive Care Unit (ICU) or the Confusion Assessment Method once a daily after surgery. The logistic regression analysis was performed for statistical analysis. In total, 172 (19.3%) patients developed POD and 151 (87.8%) patients developed POD within post-operative 3 days. Low pre-operative Hb concentration [*p* = 0.026, odds ratio (OR) = 0.978] and significant change in Hb concentration (*p* = 0.006, OR = 1.033) were significantly associated with POD. After excluding change in Hb concentration or pre-operative Hb concentration, neither of them was significantly associated with POD (*p* > 0.05). The interaction analysis of change in Hb concentration and pre-operative Hb concentration in the logistic regression model was negative. There was no significant relationship between post-operative Hb concentration and POD. Age (*p* < 0.001, OR = 1.072), stroke history (*p* = 0.003, OR = 2.489), post-operative ICU transfer (*p* = 0.007, OR = 1.981), and visual analog scale score within post-operative 2 days (*p*_1_ = 0.016 and *p*_2_ = 0.006) were independently associated with POD in the logistic regression analysis. Patients with low pre-operative Hb concentrations and high changes in Hb concentration seem to have an increased risk of POD and should receive more attention. Old age, stroke history, post-operative ICU transfer, and pain within post-operative 2 days were significantly associated with POD.

## Introduction

Post-operative delirium (POD), with a prevalence of up to 62% (1), is common in elderly patients with femoral neck fracture (FNF). POD is characterized by acute impairment of attention and cognitive function post-operatively, with fluctuating courses and various clinical manifestations, involving multiple phenotypes such as apathy, agitation, irritability, and mania (2, 3). Previous studies have shown that POD is related to short-term and long-term adverse outcomes such as prolonged hospital stay, increased mortality rate, and declined quality of life (2, 4–6). However, the pathogenesis of POD remains unclear (7), and lowering the risk of POD is considered the optimal method to decrease its incidence.

Perioperative low hemoglobin (Hb) concentrations are also prevalent in elderly patients undergoing FNF surgery and lead to prolonged length of stay (LOS) and increased risk of 30-day readmission and long-term mortality (8–10). Most studies on the relationship between perioperative Hb concentrations and POD have indicated that low pre-operative Hb concentrations would lead to a high risk of POD (11–14), whereas Myint et al. conducted an observational study of 653 patients and found no association between them (15). In all the cases, FNF surgery is associated with substantial blood loss. Although red blood cell (RBC) transfusions are performed, if needed, delayed transfusion and severe blood loss would lead to significant differences in pre-operative and post-operative Hb concentrations.

Pre-operative and post-operative Hb concentrations were determined in this study to explore whether Hb concentration and change in Hb concentration were associated with POD in elderly patients undergoing FNF surgery. In addition, the risk factors for POD were assessed.

## Materials and Methods

This single-center, retrospective, case–control study was approved by the Research and Ethics Committee of the Affiliated Hospital of Qingdao University on January 27, 2021 (registration number: QYFY WZLL 26226). Written informed consent was not obtained because of the retrospective nature of this study. This study included all the patients aged 65 years and older who had received elective FNF surgery at the Affiliated Hospital of Qingdao University from January 2016 to December 2020. The medical records of all the patients included in this study were obtained from the electronic database of the Affiliated Hospital of Qingdao University. This study also followed the Strengthening the Reporting of Observational Studies in Epidemiology (STROBE) reposting guidelines (16).

Patients were excluded based on the following criteria: (1) conservative treatment, (2) bilateral surgery or non-FNF surgery, (3) multiple traumas or fractures, (4) lack of data on Hb concentrations or blood transfusion and lack of cognitive or delirium assessment, and (5) cognitive impairment pre-operatively.

**Figure 1:**
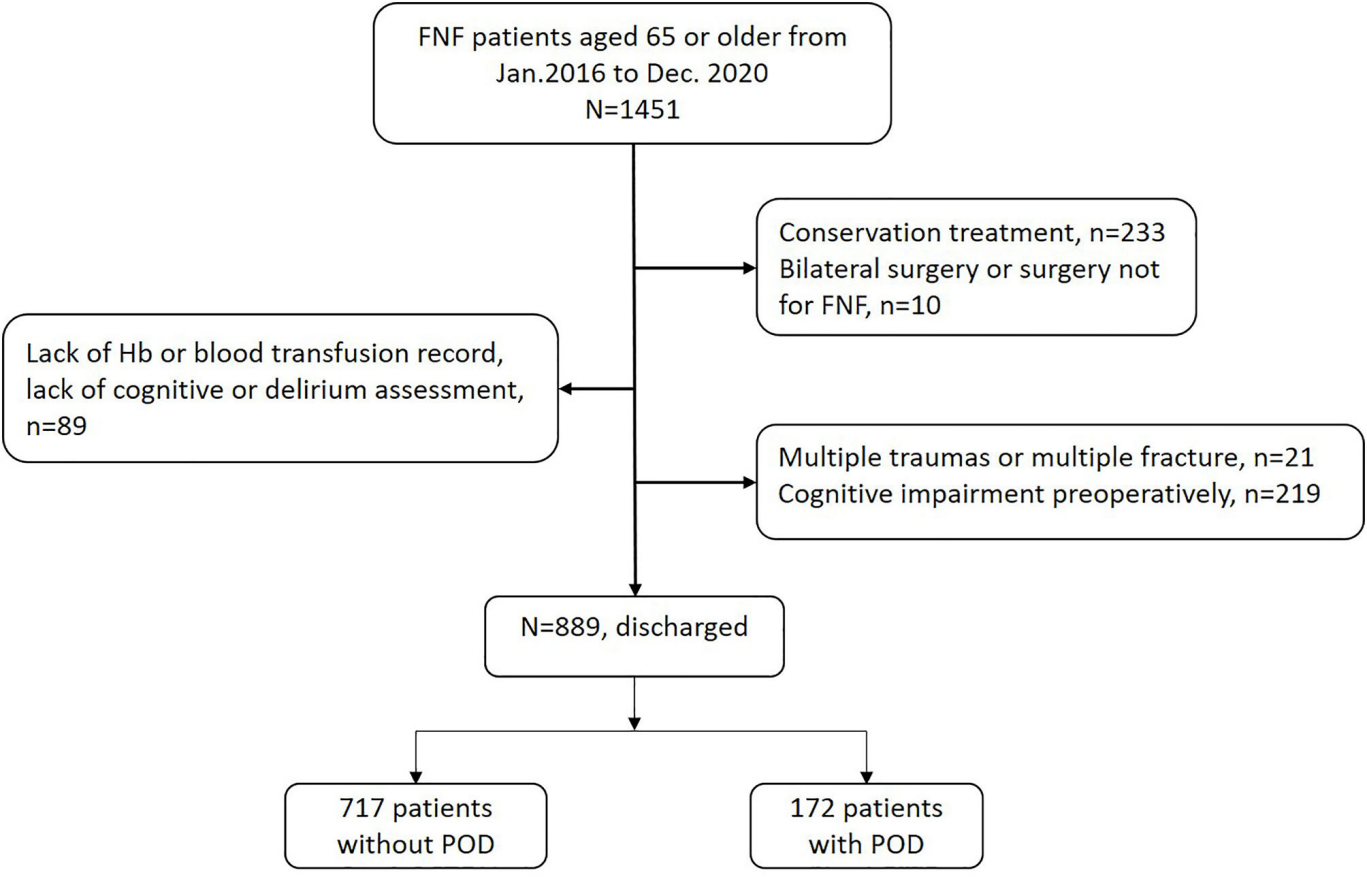
Flowchart of the case selection. FNF, femur neck fracture; Hb, hemoglobin.

Data collection was conducted by two anesthetists (HH and JG) who were blinded to the study design and statistical analysis. A total of 889 patients who met the criteria were included in the final analysis (Figure 1).

### Outcomes

The Confusion Assessment Method for the Intensive Care Unit (CAM-ICU) (17) and the CAM (18) were used to assess delirium once daily for post-operative 5 days by nurses in the ICU and ward. Both CAM and CAM-ICU contain 4 criteria: acute onset of fluctuating course, inattention, disorganized thinking, and altered consciousness, while satisfying criteria 1 and 2 and either 3 or 4, it was positive. Meanwhile, HH and JG reviewed the medical records of each patient in detail, found patients whose symptom description matched the Diagnostic and Statistical Manual of Mental Disorders-5 (DSM-5) (3) through a chart review, and confirmed the diagnosis when they had the same judgments. In patients older than 65 years, the anesthesiologist in the institution routinely assessed cognitive function using the Mini-Mental State Examination (MMSE) (19) during pre-operative visits; if the patient had the MMSE score of <27, it was recorded as having mild cognitive impairment. Finally, patients without mild cognitive impairment were categorized into the POD and non-POD groups.

### Variables

Laboratory Hb concentrations were monitored at admission and on post-operative day 1 according to the diagnostic procedure and, if necessary, according to the clinical judgment about re-examination. In this study, we extracted Hb concentrations at admission and on post-operative day 1. The change in Hb concentration was the absolute value of the difference in pre-operative and post-operative Hb concentrations.

Demographic characteristics including sex, age, body mass index, smoking and drinking history, and the American Society of Anesthesiology (ASA) score were collected. Comorbidity burden included major heart disease, lung disease, metabolic disease, and cerebrovascular disease. Hypoalbuminemia was defined as an albumin level of 35 g/l. The units of RBC transfusion from admission to post-operative 5 days were collected. Data on the operation and anesthesia time, intraoperative blood loss, types of operation and anesthesia, and post-operative ICU transfer were collected. Primary acute post-operative complications such as hypoxemia, electrolyte imbalance, and arrhythmia were collected. In addition, information on perioperative drug use and LOS was collected.

Patients were encouraged to stand and walk gradually from post-operative day 1 or mechanically-assisted rehabilitation was performed in bed. Post-operative pain assessment using the visual analog scale (VAS) was conducted by a trained nurse once daily within post-operative 3 days. The pain score was divided into four grades according to pain intensity: painless (0 point), mild (1–3 points), moderate (4–6 points), and severe (7–10 points) pain.

### Statistical Analysis

Regarding statistical analysis, categorical variables are expressed as frequencies and percentages, normally distributed continuous variables are expressed as mean and SD, and non-normally distributed continuous variables are expressed as median and interquartile range. With respect to continuous variables, Student's *t*-test was performed to test for differences between groups. The chi-squared or the Wilcoxon signed-rank tests were performed to detect differences between groups for discontinuous variables or non-normally distributed continuous variables. Patients with missing records of Hb concentrations, blood transfusion, delirium, or cognitive assessment were excluded.

Age, stroke history, pre-operative and post-operative Hb concentrations, perioperative changes in Hb concentrations, intraoperative blood loss, surgery and anesthesia type, surgery and anesthesia time, pre-operative and post-operative hypoalbuminemia, post-operative ICU transfer, post-operative hypoxemia, and the VAS scores within post-operative 3 days were analyzed using the multivariate logistic regression model. Sensitivity analyses were performed using the multivariate logistic regression with the variables deleted. The product term of pre-operative Hb concentration and change in Hb concentration [Hb (pre) × Hb change] reflects the interaction in the logistic regression analysis. Therefore, we conducted 4 models: *model 1*: logistic regression analysis contained Hb (pre- and post-operative), perioperative Hb change, age, stroke history, surgery and anesthesia type, hypoalbuminemia (pre- and post-operative), midazolam, intraoperative blood loss, operation time, anesthesia time, ICU transfer, hypoxemia, and VAS in 1–3 days after surgery; *model 2*: logistic regression analysis contained factors in model 1, except perioperative Hb change; *model 3*: logistic regression analysis contained factors in model 1, except pre-operative Hb concentration; *model 4*: logistic regression analysis contained factors in model 1 and factor “Hb (pre) × Hb change.” Statistical significance was set at a two-tailed *p*-value of 0.05. Data were coded and stored and analyzed using the Statistical Package for the Social Sciences (SPSS) version 22.0 (SPSS Incorporation, Chicago, Illinois, USA).

## Results

### Incidence of POD and Baseline Characteristics

A total of 172 patients (19.3%) developed POD. Of these patients, 151 patients (87.8%) developed delirium within post-operative 3 days (Table 1). The mean age in the POD and non-POD groups was 81.3 ± 7.0 and 77.3 ± 7.3 years, respectively. A total of 646 women (72.7%) were finally included. In this study, 732 patients (82.3%) had the ASA 3 grade and no patient had the ASA 1 grade in this study. LOS was longer in the POD group than in the non-POD group (11.3 ± 5.1 vs. 10.5 ± 4.7 days, *p* = 0.078) (Table 2). No patient data was lost from the 889 participants. No patient was painless on the first day after surgery both in the POD and non-POD groups.

**Table 1:** The time of post-operative delirium happening from surgery end.

**Days after surgery**	**0**	**1st**	**2nd**	**3rd**	**4th**	**5th**
Frequency, *n*	4	69	46	32	11	10
Percentage, %	2.3	40.1	26.7	18.6	6.4	5.8

**Table 2:** The univariate analysis of post-operative delirium.

	**Without POD (*n* = 717)**	**POD (*n* = 172)**	***P*-value**
Pre-operative hemoglobin, g/l	120.5 ± 16.6	116 ± 16.4	**0.004**
Post-operative hemoglobin, g/l	101.1 ± 15.9	96.2 ± 16.2	**<0.001**
Perioperative hemoglobin change, g/l	20.7 ± 12.2	23.1 ± 12.8	**0.023**
**Physical characteristics**			
Age, years	77.3 ± 7.3	81.3 ± 7.0	**<0.001**
Female, *n*	525 (73.2%)	121 (70.3%)	0.448
BMI, kg/m^2^	22.9 ± 3.7	22.8 ± 3.8	0.869
Smoking history	86 (12.0%)	19 (11.0%)	0.729
Drinking history	68 (9.5%)	17 (9.9%)	0.873
**ASA status**			
2	78 (10.9%)	12 (7.0%)	0.187
3	586 (81.7%)	146 (84.9%)	
4	53 (7.4%)	14 (8.1%)	
Hypertension on medicine	271 (37.8%)	68 (39.5%)	0.673
Myocardial infarction	10 (1.4%)	1 (0.6%)	0.629
Coronary heart disease	98 (13.7%)	21 (12.2%)	0.614
Atrial fibrillation	18 (2.5%)	7 (4.1%)	0.393
Diabetes mellitus	145 (20.2%)	27 (15.7%)	0.177
Stroke history	53 (7.4%)	22 (12.8%)	**0.022**
COPD	29 (4.0%)	8 (4.7%)	0.721
Parkinson's disease	12 (1.7%)	2 (1.2%)	0.887
**Operative values**			
**Surgery type**			
Total hip replacement	433 (60.4%)	81 (47.1%)	**0.005**
Hemiarthroplasty	169 (23.6%)	58 (33.7%)	
Internal fixation	115 (16.0%)	33 (19.2%)	
Anesthesia type, intraspinal	297 (41.4%)	59 (34.3%)	**0.087**
Hypoalbuminemia (pre)	101 (14.1%)	36 (20.9%)	**0.026**
Hypoalbuminemia (post)	386 (53.8%)	116 (67.4%)	**0.001**
Opioid use	452 (63.0%)	118 (68.6%)	0.172
Midazolam use	169 (23.6%)	54 (31.4%)	**0.034**
Benzodiazepines use	24 (3.3%)	8 (4.7%)	0.410
Blood transfusion, IU	2 (2–3)	2 (2–3)	0.286
Intraoperative blood loss, ml	200 (200–300)	200 (200–300)	**0.063**
Operation time, min	89.1 ± 30.7	97.6 ± 40.3	**0.002**
Anesthesia time, min	123.1 ± 34.4	128.3 ± 34.8	**0.020**
**Post-operative values**			
ICU transfer	147 (20.5%)	66 (38.4%)	**<0.001**
Hypoxemia	1 (0.1%)	2 (1.2%)	**0.097**
Electrolyte disturbance	22 (3.1%)	6 (3.5%)	0.777
Arrhythmia	5 (0.7%)	2 (1.2%)	0.889
**VAS 1 day after surgery**			**<0.001**
Mild	714 (99.6%)	155 (90.1%)	
Moderate	3 (0.4%)	17 (9.9%)	
**VAS 2 day after surgery**			**<0.001**
Painless	86 (12.0%)	10 (5.8%)	
Mild	628 (87.6%)	146 (84.9%)	
Moderate	3 (0.4%)	16 (9.3%)	
**VAS 3 day after surgery**			**<0.001**
Painless	409 (57.0%)	72 (41.9%)	
Mild	308 (43.0%)	100 (58.1%)	
LOS, days	10.5 ± 4.7	11.3 ± 5.1	**0.078**

*POD, post-operative delirium; Hb, hemoglobin; OR, odds ratio; BMI, body mass index; ASA, American Society of Anesthesiology; COPD, chronic obstructive pulmonary disease; ICU, intensive care unit; VAS, visual analog scale; LOS, length of stay*.

### Hemoglobin and the Hb Change and POD

The pre-operative and post-operative Hb concentrations were lower in the POD group than in the non-POD group (116 ± 16.4 vs. 120.5 ± 16.6 g/l, *p* = 0.004; 96.2 ± 16.2 vs. 101.1 ± 15.9 g/l, *p* < 0.001). No patient had Hb concentration of <7 g/l pre-operatively, whereas 22 patients (2.5%) had post-operative Hb concentration of <7 g/l. The mean change in Hb concentration was 21.2 ± 12.3 g/l and the change in perioperative Hb concentration was higher in the POD group than in the non-POD group (23.1 ± 12.8 vs. 20.7 ± 12.2 g/l).

Low pre-operative Hb concentrations [*p* = 0.026, odds ratio (OR) = 0.978, 95% CI = 0.959–0.997] and change in perioperative Hb concentration (*p* = 0.006, OR = 1.033, 95% CI = 1.010–1.057) together were significantly associated with POD after adjustment for age, stroke history, surgery and anesthesia type, hypoalbuminemia (pre-operatively and post-operatively), midazolam use, intraoperative blood loss, operation and anesthesia time, post-operative ICU transfer and hypoxemia, and the VAS score within post-operative 3 days (Table 3). After excluding one of the two factors (pre-operative Hb concentration and change in Hb concentration), respectively, with other confounding factors left unchanged, another factor was no more significant (Table 4). Including the factor “Hb (pre) × Hb change” in the logistic regression analysis, there was no interaction effect between pre-operative Hb concentration and the change of Hb concentration. Moreover, the effect of the factor “Hb change” was significant in *model 4* (*p* = 0.022, OR = 1.139, 95% CI = 1.019–1.274), while the effect of “Hb (pre)” was not.

**Table 3:** The logistic analysis of POD.

	***P*-value**	**OR (95% CI)**
Hb (pre)	**0.026**	0.978 (0.959–0.997)
Hb (post)	0.388	1.009 (0.988–1.030)
Perioperative Hb change	**0.006**	1.033 (1.010–1.057)
Age	**<0.001**	1.072 (1.041–1.104)
Stroke history	**0.003**	2.483 (1.374–4.489)
**Surgery type**	0.498	
Hemiarthroplasty/total hip replacement		0.754 (0.439–1.294)
Internal fixation/total hip replacement		0.744 (0.408–1.355)
Anesthesia type	0.600	1.135 (0.707–1.823)
Hypoalbuminemia (pre)	0.988	0.996 (0.593–1.672)
Hypoalbuminemia (post)	0.419	1.203 (0.768–1.882)
Midazolam use	0.140	1.387 (0.898–2.144)
Intraoperative blood loss	0.581	1.001 (0.998–1.004)
Operation time	0.168	1.009 (0.996–1.023)
Anesthesia time	0.171	0.992 (0.980–1.004)
ICU transfer	**0.007**	1.981 (1.205–3.258)
Hypoxemia	0.186	5.495 (0.441–68.522)
VAS 1 day after surgery	**0.016**	5.937 (1.386–25.431)
**VAS 2 day after surgery**	**0.006**	
Mild/painless		1.692 (0.810–3.537)
Moderate/painless		15.551 (2.931–82.138)
VAS 3 day after surgery	0.217	1.281 (0.864–1.899)

*After adjustment for Hb (pre- and post-operative), perioperative Hb change, age, stroke history, surgery and anesthesia type, hypoalbuminemia (pre), hypoalbuminemia (post), midazolam, intraoperative blood loss, operation time, anesthesia time, ICU transfer, hypoxemia, and the VAS in 1–3 days after surgery*.

*POD, post-operative delirium; Hb, hemoglobin; OR, odds ratio; ICU, intensive care unit; VAS, visual analog scale*.

**Table 4:** Different models of the logistic analysis of the independent risk factors (*p* < 0.05) of POD.

	**Model 1**	**Model 2**	**Model 3**	**Model 4**
	***P*-value**	***P*-value**	***P*-value**	***P*-value**
Hb (pre)	0.026	0.645	/	0.983
Perioperative Hb change	0.006	/	0.087	0.022
Hb (pre) * Hb change	/	/	/	0.079
Age	<0.001	<0.001	<0.001	<0.001
Stroke history	0.003	0.004	0.004	0.002
ICU transfer	0.007	0.008	0.006	0.006
VAS 1 day after surgery	0.016	0.008	0.008	0.021
VAS 2 day after surgery	0.006	0.007	0.007	0.004

**Figure 2:**
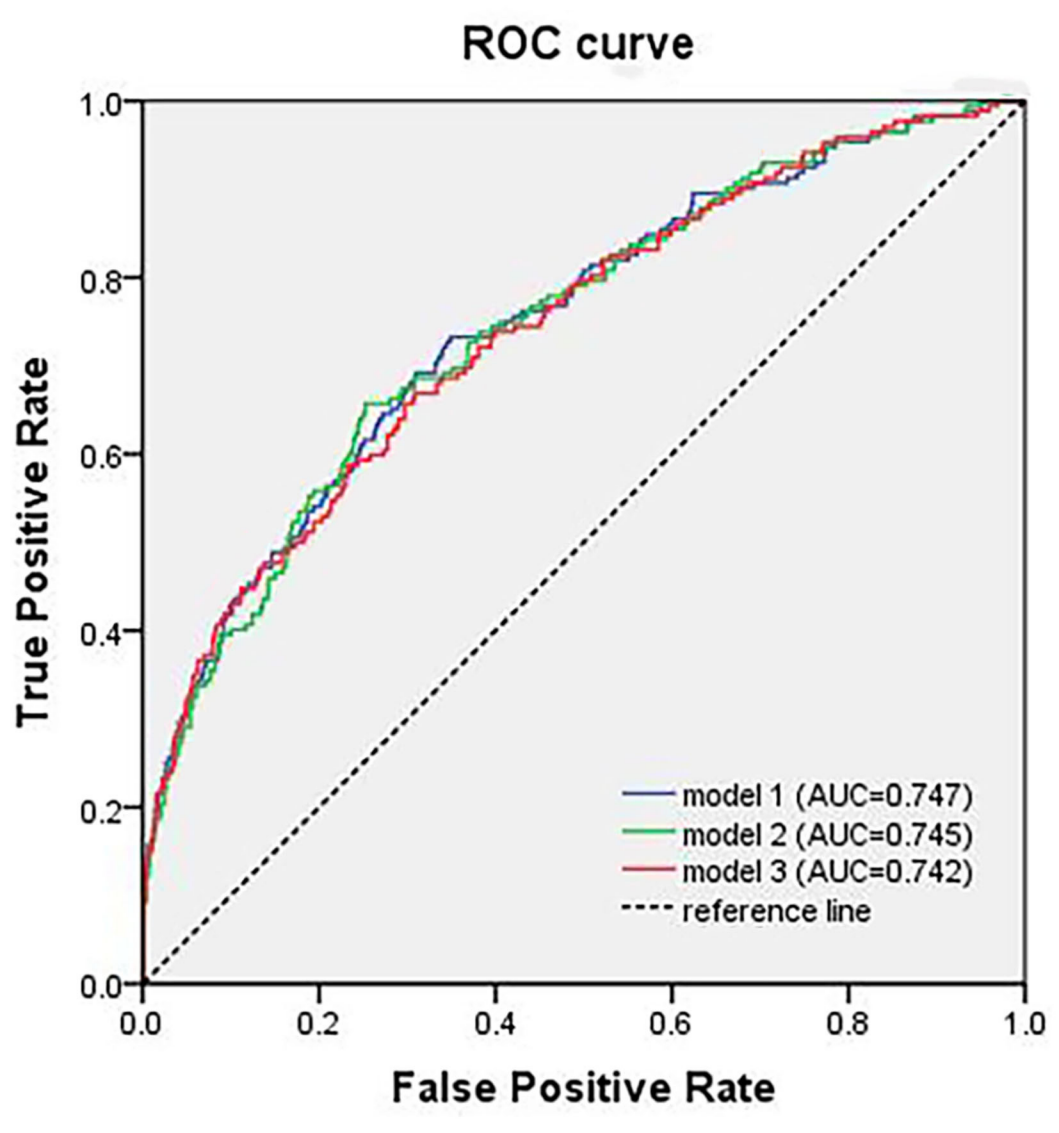
The ROC curve analysis of different models of the logistic analysis. ROC, receiver operating characteristic; AUC, area under the curve.

The difference of the area under the curve (AUC) between models, which included pre-operative Hb concentration and Hb change, respectively, in the receiver operating characteristic (ROC) curve analysis, was fairly small (*model 2*: AUC = 0.745, *p* < 0.001, 95% CI = 0.703–0.787; *model 3*: AUC = 0.742, *p* < 0.001, 95% CI = 0.699–0.784) (Figure 2). Age (*p* < 0.001, OR = 1.072), stroke history (*p* = 0.003, OR = 2.489), post-operative ICU transfer (*p* = 0.007, OR = 1.981), and the VAS score within post-operative 2 days (*p*_1_ = 0.016 and *p*_2_ = 0.006) were independently associated with POD in the logistic regression analysis.

## Discussion

In this case–control study, pre-operative Hb concentration and perioperative change in Hb concentration were significantly associated with POD and perioperative change in Hb concentration seemed to be more important. Advanced age, stroke history, ICU transfer, and the VAS score within post-operative 2 days were found to be significant independent risk factors for POD.

Many studies have focused on the relationship between Hb concentrations and POD (13, 20). The association between low pre-operative Hb concentration and POD is consistent with the results of previous studies. Tahir et al. conducted a prospective observational study of 381 patients and found that low Hb concentration might increase the risk of POD by ~3-fold (21). Elsamadicy et al. conducted a retrospective cohort study and found that pre-operative lower Hb concentrations were significantly associated with POD (13). One of the most popular plausible explanations is that low Hb concentrations reduce cerebral oxygen delivery (7, 22), which is also one of the theories of pathophysiology of POD. We found no statistical significance between post-operative Hb concentration and POD. This result might be related to routine oxygen inhalation for 12 h after surgery in our medical center. The routine post-operative oxygen treatment might have effectively prevented the occurrence of tissue hypoxia, so it also reduced the occurrence of POD. However, whether improving hypoxia would prevent POD was beyond the aim of this study and should be further studied. The results in this study suggested that not only low Hb concentration should be corrected in elderly patients perioperatively, but a great perioperative change in Hb concentration should be avoided to reduce the occurrence of POD.

To the best of our knowledge, this is the first study to find that perioperative change in Hb concentration was associated with POD and after interaction analysis, it might be more important. We hypothesize that the perioperative change in Hb concentration might contribute to POD by reducing the oxygen delivery of the brain and further studies are needed to demonstrate it. The mean change in Hb concentration in this study was 21.2 ± 12.3 g/l; this is consistent with the finding of a previous observational study (8), which involved 1,534 patients and found that the mean perioperative change in Hb concentration was 19–30 g/l. Though the change in Hb concentration was significantly related to POD, the OR indicated that the influence was small. This might be due to many factors that can affect Hb concentration. Perioperative blood loss is the most important factor for the change in Hb concentration. In all cases, FNF surgery results in a large amount of blood loss (23), and post-operative blood loss is ~1.6-fold of intraoperative blood loss. We found that detailed records of post-operative blood loss were lacking; therefore, only intraoperative blood loss was recorded. In addition, pre-operative hemoconcentration and post-operative hemodilution might contribute to change in Hb concentrations. In an observational prospective study of 225 patients, Clemmesen et al. (24) found that only three patients had low Hb concentrations at admission, whereas the majority of patients had low Hb concentrations at the time of surgery. This indicated that pre-operative hemoconcentration might be common in patients undergoing FNF surgery. Pre-operative Hb concentration is often inflated due to hypovolemia and/or dehydration, masking the actual low Hb concentration. Therefore, fluid replacement or transfusion therapy could be important. However, it is hard for us to identify the relationship between them and POD for the lack of pre-operative records.

The prevalence of POD varied from 10.5 to 42.0% in previous studies (25–29). The incidence of POD in this study was 19.3%, which is consistent with the results of previous studies. Older age, stroke history, and post-operative ICU transfer were found to be significantly associated with POD and these were generally clarified in previous studies (7, 30). The finding that the VAS score in post-operative 2 days was significantly associated with POD is consistent with those of previous studies, which showed that pain was an independent post-operative risk factor for POD (31–34). This result also indicated that analgesia efficiency was not good, even for patients in our center for whom a multimodal analgesia strategy was used for post-operative pain. Therefore, analgesia strategies should be investigated in further studies.

### Strengths and Limitations

This study has some strengths. The sample size of 889 patients was large. As far as we know, this study is the first study to assess the relationship between change in Hb concentration and POD; This study found a positive association between the two.

This study has some limitations. First, this was a retrospective case–control study, information was obtained from medical records, and the strength of the causal relationship was limited. Second, the once daily assessment of POD in this study might increase the missed diagnosis and underestimate the incidence of POD. Given that, our researchers reviewed the medical records of each patient in detail, found patients whose symptom description matched POD through the chart review, and as supplements only when the two reviewers confirmed the diagnosis in agreement. Moreover, the medical records of perioperative blood loss and fluid replacement were incomplete. We only collected data on intraoperative blood loss, the data on post-operative blood loss, and the perioperative volume of fluid that could not be obtained accurately. Therefore, the relationship between perioperative blood loss and the perioperative fluid volume and POD needs to be verified in further studies. In addition, this was a single-center study. Therefore, the generalizability of the results is limited. Large-scale multicenter studies are needed in the future.

In conclusion, the results of this study showed that pre-operative Hb concentration and perioperative changes in Hb concentration were significantly associated with POD. Age, stroke history, post-operative ICU transfer, and the VAS score in post-operative 2 days were independently associated with POD.

## Data Availability Statement

The original contributions presented in the study are included in the article/Supplementary Material, further inquiries can be directed to the corresponding author/s.

## Ethics Statement

The studies involving human participants were reviewed and approved by the Research and Ethics Committee of the Affiliated Hospital of Qingdao University. Written informed consent for participation was not required for this study in accordance with the national legislation and the institutional requirements.

## Author Contributions

Y-ML contributed to the study design, data analysis, and performed the manuscript. HH and JG selected suitable patients from the database and extracted information of patients and filtered it. JZ contributed to the study design and data analysis. H-CC conceived the study design and revised the manuscript for submission. All authors read and approved the final manuscript.

## Conflict of Interest

The authors declare that the research was conducted in the absence of any commercial or financial relationships that could be construed as a potential conflict of interest.

## Publisher's Note

All claims expressed in this article are solely those of the authors and do not necessarily represent those of their affiliated organizations, or those of the publisher, the editors and the reviewers. Any product that may be evaluated in this article, or claim that may be made by its manufacturer, is not guaranteed or endorsed by the publisher.
